# The impact of COVID-19 on the Child and Adolescent Psychiatric Emergency Departments of the paediatric university hospital in Brussels

**DOI:** 10.1192/j.eurpsy.2023.1219

**Published:** 2023-07-19

**Authors:** S. Marchini, B. Tedik, C. Bennis, V. Delvenne

**Affiliations:** 1 Department of Psychiatry for Infant, Child, Adolescent and Youth, University Hospital of Brussels (HUB); 2 Faculty of Medicine, Université Libre de Bruxelles (ULB), Brussels; 3 Department of Adolescent Psychiatry, Le Domaine hospital, Braine-l’Alleud, Belgium

## Abstract

**Introduction:**

Since the COVID-19 pandemic outbreak, children and adolescents mental health has been severely impacted. During the first lockdown measures in the spring of 2020, we observed a decrease in access to care, principally due to the “stay home” policy. From the beginning of the year 2021, we observed a massive increase in admission to the Child and Adolescent Psychiatry (CAP) Emergency Departments (ED) (Beaudry *et al.* Ann. Gen. Psychiatry 2022; 21(1), 17; Hernández-Calle *et al*. SJCAPP 2022; 10(1), 53–57).

**Objectives:**

First, this study aimed to quantify the increase in patients’ admissions to the CAP ED of the paediatric university hospital in the urban area of Brussels. Second, we described the reasons for admission.

**Methods:**

We conducted an observational retrospective study in the French-speaking Belgium paediatric university hospital in Brussels. Through the ED register, we selected all the admissions for psychiatric reasons from 1^st^ December 2020 to 1^st^ December 2021. Data were collected from patients’ electronic medical records and compared to data from 1^st^ January 2013 to 29^th^ February 2016, already collected in 2016 from the same ED. To compare the variables between 2013-2016 and 2020-2021, we performed a Student’s t-test for the number of admissions to CAP EP, a chi-squared test for sex rate, suicide attempts and urgent hospitalizations, and a Wilcoxon test for the median age. The study protocol was approved by the Queen Fabiola Children’s University Hospital IRB (reference 99/21).

**Results:**

Female patients were more represented in 2020-2021 than in 2013-2016 (48% compared to 66.1%, *p<0.001*), and the median age increased from 12 to 14 y.o. (*p<0.05*). Compared to a few years before, we registered a 280.79% increase in the admissions to the CAP ED, resulting in 252 admissions in 38 months (2013-2016) compared to 303 in 12 months (2020-2021) (*p<0.001*). Admissions for suicidal attempts were twice higher than 2013-2016 (from 6.8% to 12.5%; *p<0.001*) and urgent hospitalizations increased eight times (from 0.8% to 6.7%; *p<0.001*). Admissions to the CAP ED started to decrease progressively during the whole year 2021, with the lowest peak during summer holidays (**Fig.1**). In 2013-2016, the 3 main reasons for admissions to the CAP ED were disruptive behaviors (15.1%), psychomotor agitation (14.2%) and somatic manifestations (12.3%); whereas in 2020-2021, these were suicidal ideations (14%), suicide attempts (12.5%) and somatic manifestations (10.4%).

**
Figure 1** – Admissions to the CAP ED during 2020-2021, rates per month.

**Image:**

**Figure:**
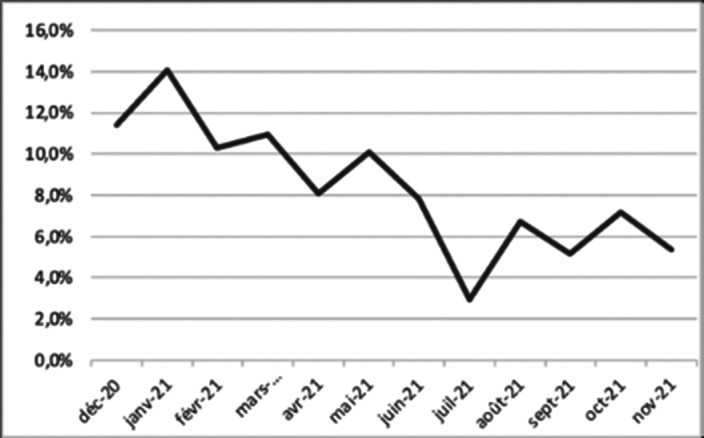

**Conclusions:**

According to our comparative study and the literature, admissions to the CAP ED increased significantly during the second wave of the COVID-19 pandemic, especially for suicidal ideations and suicide attempts. The urgent hospitalisations are eight times higher during the period 2020-2021.

**Disclosure of Interest:**

None Declared

